# Skin Electroporation: Effects on Transgene Expression, DNA Persistence and Local Tissue Environment

**DOI:** 10.1371/journal.pone.0007226

**Published:** 2009-09-30

**Authors:** Anna-Karin Roos, Fredrik Eriksson, James A. Timmons, Josefine Gerhardt, Ulrika Nyman, Lindvi Gudmundsdotter, Andreas Bråve, Britta Wahren, Pavel Pisa

**Affiliations:** 1 Department of Oncology and Pathology, Cancer Center Karolinska R8:01, Immune and Gene Therapy Laboratory, Karolinska Institute, Stockholm, Sweden; 2 Royal Veterinary College, University of London, Camden, London, United Kingdom; 3 Institute of Environmental Medicine, Toxicology and Neurotoxicology, Karolinska Institute, Stockholm, Sweden; 4 Department of Microbiology, Tumor and Cell Biology, Karolinska Institutet & Swedish Institute for Infectious Disease Control, Stockholm, Sweden; Comprehensive AIDS Reseach Center, China

## Abstract

**Background:**

Electrical pulses have been used to enhance uptake of molecules into living cells for decades. This technique, often referred to as electroporation, has become an increasingly popular method to enhance *in vivo* DNA delivery for both gene therapy applications as well as for delivery of vaccines against both infectious diseases and cancer. *In vivo* electrovaccination (gene delivery followed by electroporation) is currently being investigated in several clinical trials, including DNA delivery to healthy volunteers. However, the mode of action at molecular level is not yet fully understood.

**Methodology/Principal Findings:**

This study investigates intradermal DNA electrovaccination in detail and describes the effects on expression of the vaccine antigen, plasmid persistence and the local tissue environment. Gene profiling of the vaccination site showed that the combination of DNA and electroporation induced a significant up-regulation of pro-inflammatory genes. *In vivo* imaging of luciferase activity after electrovaccination demonstrated a rapid onset (minutes) and a long duration (months) of transgene expression. However, when the more immunogenic prostate specific antigen (PSA) was co-administered, PSA-specific T cells were induced and concurrently the luciferase expression became undetectable. Electroporation did not affect the long-term persistence of the PSA-expressing plasmid.

**Conclusions/Significance:**

This study provides important insights to how DNA delivery by intradermal electrovaccination affects the local immunological responses of the skin, transgene expression and clearance of the plasmid. As the described vaccination approach is currently being evaluated in clinical trials, the data provided will be of high significance.

## Introduction

Numerous strategies – physical, chemical and immunological – are under investigation to improve the efficacy of DNA vaccines (reviewed in [1], [2]). *In vivo* electroporation devices have proven to be effective tools for enhanced delivery of DNA to muscle, skin and tumors. A wide range of studies have demonstrated that using electroporative DNA administration in small [3], [4] as well as large [5], [6] animals greatly increases gene expression and also induces impressive immune responses. The first gene therapy product with delivery by electroporation was licensed in 2007 in Australia for use in swine [1]. Several clinical investigations of intramuscular DNA delivery by electroporation have been under way since 2004 [7] and a number of new clinical investigations of both intramuscular and intradermal electroporation are in the pipeline. Results from the first clinical study of intratumoral electroporative DNA delivery recently demonstrated that *in vivo* electroporation is safe, effective and reproducible in patients with metastatic melanoma [8].

Pre-clinical electrovaccination (gene delivery followed by electroporation) studies performed to investigate the effect of electroporation on antigen expression kinetics [4], [9], [10], DNA persistence [11]–[13], local tissue injury, inflammation and cellular infiltration [12], [14] have generally utilized intramuscular DNA delivery and electroporation. Since electroporation increases transgene expression 10-100-fold [9], [10], [15], [16], probably due to a higher cellular uptake of DNA molecules, safety-related questions have been raised about the possibility that electroporation could lead to increased DNA persistence and a higher integration frequency [7], [17]. Results from studies of intramuscular electroporation are inconclusive, showing both increased integration [13], unchanged persistence of DNA in tissue [11], [12] and decreased levels of plasmid associated with high molecular genomic DNA [11]. However, to our knowledge, no data have been published on plasmid persistence or integration after skin electroporation. Furthermore, it has been suggested that intramuscular electroporation has adjuvant-like properties, such as generation of a pro-inflammatory milieu with cytokine release, cellular infiltration and moderate tissue injury [18]–[20]. Although both cellular infiltration and tissue damage have been indicated after skin electroporation [21], there is a lack of data both on the effect of the local tissue environment at the molecular level and on the possibility of an adjuvant effect.

Skin electroporation of genetic vaccines has shown very promising immune responses in small [16], [22], [23] and large animal models [21], [24] and is a very attractive DNA delivery method for clinical use. Skin is an ideal target for DNA vaccine delivery, as it is rich in antigen-presenting cells, such as Langerhans' cells and dermal dendritic cells. It also allows uncomplicated monitoring of the vaccination area. Skin electroporation permits topical application of a local anesthetic and uses short electrode needles. This is expected to significantly improve the tolerability of vaccine delivery compared to intramuscular electroporation, especially as the electrodes will not stimulate nearly as much muscle contraction. The effects of electrovaccination in the skin need to be delineated with the same vigilance as for intramuscular electroporation.

This study investigates the functional properties of intradermal electroporative DNA delivery. It has evaluated the kinetics of transgene expression after DNA injection, the location of transfected cells in the skin, the effect on the local tissue environment and the persistence of DNA molecules at the injection site.

## Results

### Intradermal DNA electroporation results in rapid, high and stable transgene expression, predominantly in the hypodermis

**Figure 1:**
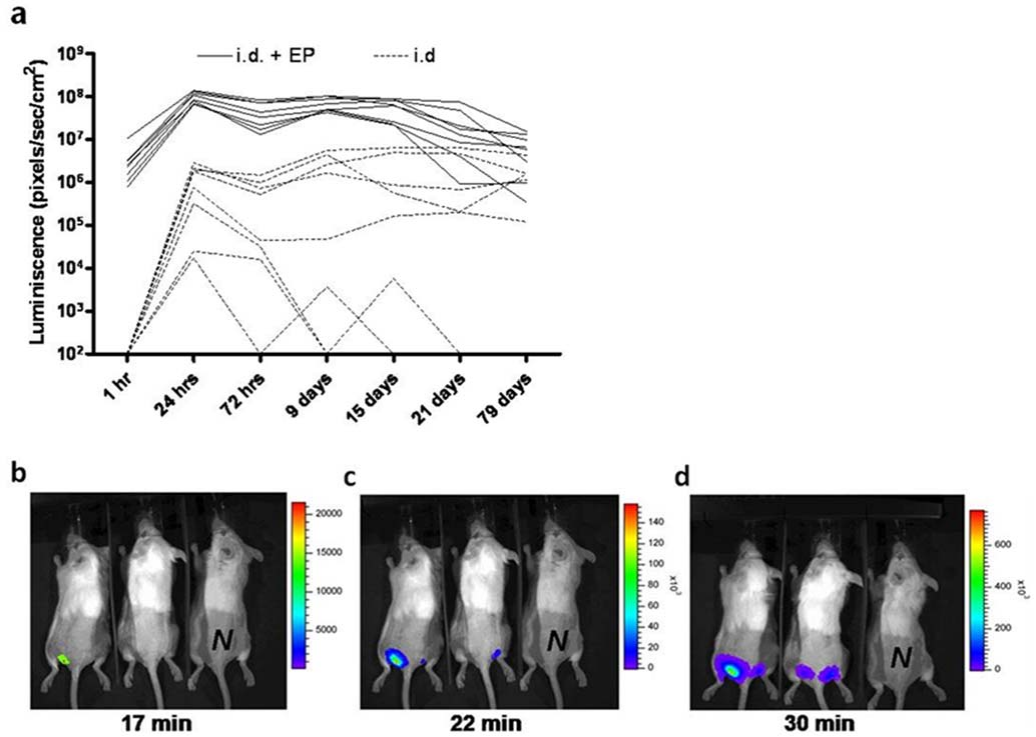
Time kinetics of transgene expression in skin after DNA electrovaccination. (a) Time course of *in vivo* luciferase expression after intradermal (i.d.) DNA delivery alone (dotted line) and after i.d. DNA delivery followed by electroporation (filled line). One representative experiment of two is shown (n = 8). (b–d) Immediate monitoring of gene expression after DNA electrovaccination. Representative bioluminescent images showing luciferase expression in skin at different time points after DNA electrovaccination. *N* denotes the negative control (non-injected). The scale shows intensity of luminescence (photons/sec/cm^2^). The experiment was repeated three times.

The impact of electroporation on transgene expression after intradermal DNA delivery was investigated. Mice received intradermal injections of 10 µg luciferase encoding plasmid and half of them were subjected to electroporation. Luciferase expression was measured using an *in vivo* imaging system. Mice receiving DNA in combination with electroporation expressed luciferase already after 1 hr (**Fig. 1a**), while expression in the non-electroporated mice was below the detection level (<100 pixels/sec/cm^2^). Luciferase expression in the electroporated mice was more stable and the average emission was up to 2 logs higher than in the non-electroporated mice. Average protein expression in the electroporated group remained significantly higher until day 15 compared to the non-electroporated mice (**Fig. 1a**). In a longer follow-up study, an average level of 10^6^ pixels/sec/cm^2^ of luminescence was detected 5 months after a single electrovaccination of 10 µg DNA (data not shown). After the observation that electrovaccinated mice had detectable luciferase expression already 1 hr after vaccination (**Fig. 1a**) the early kinetics of luciferase gene expression after electrovaccination was investigated in greater detail. Bioluminescent images showed that the luciferase gene was expressed as early as 17 minutes after DNA injection (**Fig. 1b**). At 22 minutes post injection, 3 out of 4 injection sites expressed the luciferase gene (**Fig. 1c**) and all sites did so after 30 minutes (**Fig. 1d**).

**Figure 2:**
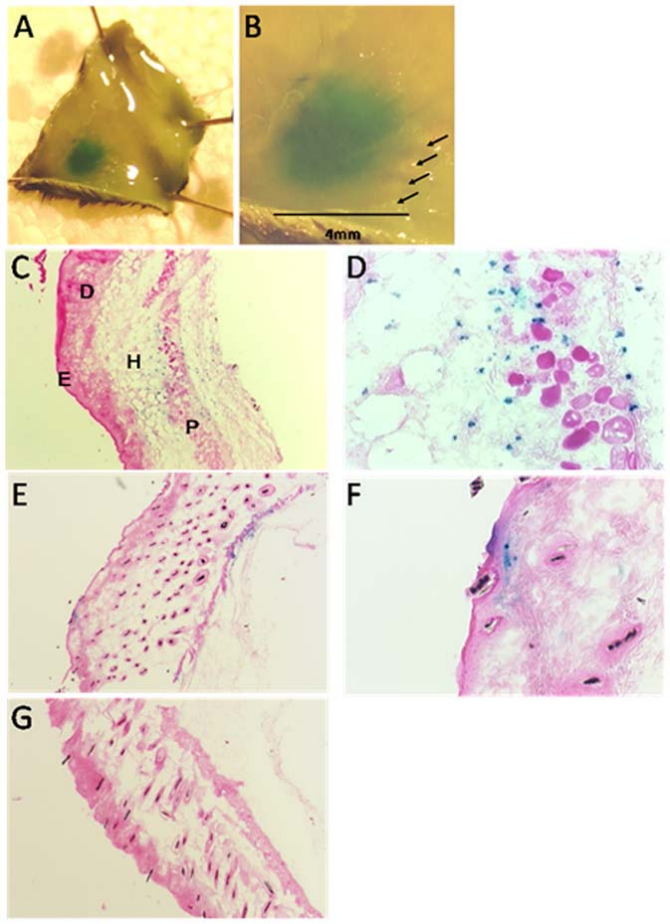
β-galactosidase expression in skin following DNA electrovaccination. Mice were injected intradermally with 20 ug DNA encoding LacZ and the injection site was electroporated. (a, b) Skin biopsies were removed after 24 hrs and stained with X-gal (a); arrows indicate needle penetration sites (b). (c–g) Distribution of β-galactosidase expressing cells in skin sections 24 hrs after DNA electrovaccination. Skin sections were prepared for histochemistry and stained with X-gal; 4X (c, e, g) and 20X (d, f) magnifications of skin sections show β-galactosidase expressing cells around the panniculus carnosus muscle layer (c–d) and in the hypodermis (c–e), dermis and epidermis (e, f). Untreated skin stained with X-gal was used as a negative control (g). P = panniculus carnosus, H = hypodermis, D = dermis and E = epidermis.

As *in vivo* monitoring of luciferase expression does not show individual cells and the light emission spreads beyond the location of transfected cells, histochemical staining of β-galactosidase was used to investigate the location of the DNA transfected cells in the skin. This demonstrated that the use of needle array electrodes produced a uniform transfection of the treated volume, resulting in a large area of transfected cells confined to the space between the needle rows (**Fig. 2a**), with the majority of the transfected cells concentrated around the panniculus carnosus muscle layer in the deepest layer of the skin (**Fig. 2c–e**). Transfected cells were also found in the hypodermis (**Fig. 2c–e**) and in sparse numbers through the dermis and epidermis (**Fig. 2e, f**). However, no expression was observed in actual muscle cells. No background staining was seen in untreated skin (**Fig. 2g**) and neither luciferase nor LacZ expression was detected in the tissue immediately below the injected skin (data not shown). The data demonstrate that intradermal DNA delivery in combination with electroporation results in cell transfection throughout all layers of the skin and yields faster, higher and more consistent gene expression, compared to intradermal gene delivery alone.

### Co-injection of an immunogenic antigen abolishes long-term luciferase expression

**Figure 3:**
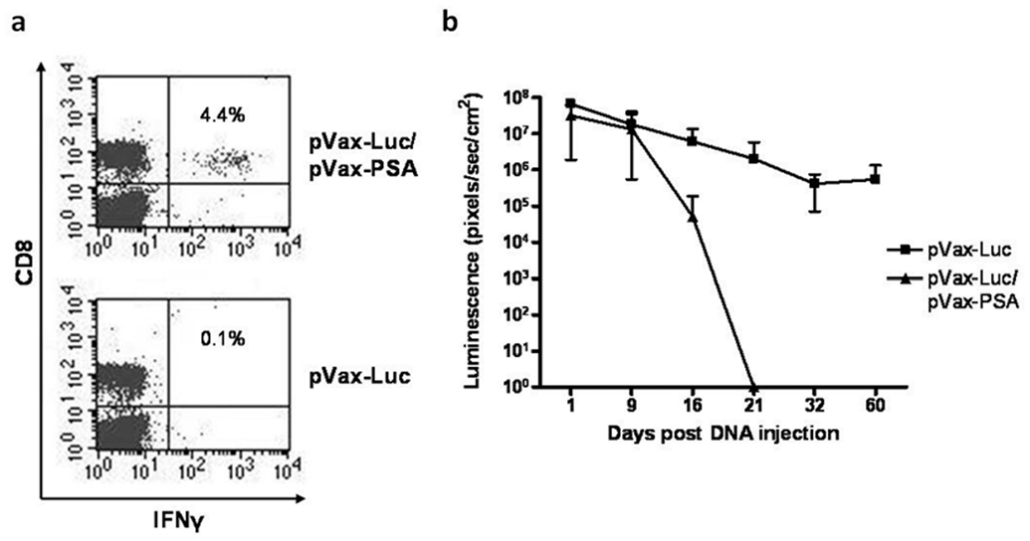
Effect of vaccine-specific T cells on transgene expression in skin. Mice were vaccinated with a combination of pVax-Luc/pVax-PSA or pVax-Luc alone and were then analyzed for PSA-specific T cells and luciferase expression. (a) Representative flow cytometry dot plots showing interferon-γ producing CD8^+^ T cells (upper right quadrant) in response to PSA peptide stimulation, pVax-Luc/pVax-PSA vaccination (upper panel) and pVax-Luc vaccination (lower panel). (b) Time course of luciferase expression in skin. pVax-Luc alone (filled squares) or in combination with pVax-PSA (filled triangles). Shown is mean±SD (n = 9). The experiment was repeated three times.

To test whether the observed long-term expression of luciferase is associated with its relatively low immunogenicity [25], the pVax-luc plasmid was co-injected with plasmid pVax-PSA. The hypothesis was that co-injection with prostate specific antigen (PSA), which is highly immunogenic in mice [16], would cause a T cell mediated immune attack on the PSA/luciferase transfected cells and therefore result in elimination of luciferase expressing cells. To confirm that PSA-specific CD8^+^ T cells were induced by the vaccination, blood was collected from the tail of mice after two weeks and then the mice were kept for further luminescence monitoring. In mice vaccinated with a combination of pVax-luc and pVax-PSA, an average of 3.7±0.7% (SD) of the CD8^+^ T cells produced IFN-γ in response to stimulation with a PSA-derived peptide (**Fig. 3a**). No PSA-specific T-cells were detected when electroporation was applied seconds before DNA injection (**supporting information Fig. S1**). The background response of T cells isolated from mice vaccinated with pVax-luc was less than 0.1% (**Fig. 3a**). *In vivo* imaging analysis revealed that luciferase gene expression did not differ between the groups during the first 9 days after vaccination (**Fig. 3b**). At day 16, luciferase expression had significantly dropped in mice co-injected with the PSA expressing plasmid, following the appearance of PSA-specific CD8^+^ cells, and at day 21 the expression was below detection level (**Fig. 3b**). In mice vaccinated with only pVax-luc, protein expression was still high 60 days after vaccination (**Fig. 3b**). This suggests that electrovaccination with an immunogenic antigen can induce a cellular immune response capable of eliminating transgene transfected cells.

### Electroporation after intradermal DNA injection does not result in increased plasmid persistence

**Figure 4:**
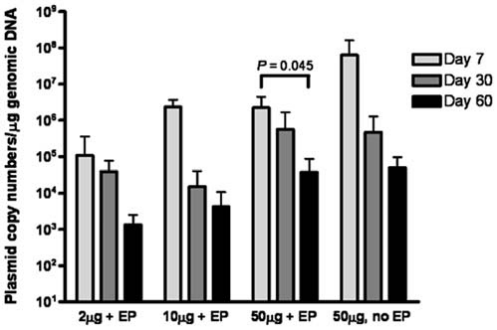
Plasmid persistence in skin over time. Histogram showing numbers of persisting plasmid copies at the indicated time-points after intradermal DNA injection of the specified doses. Error bars represent mean±SD (*n* = 6 individual samples). Each individual sample was analyzed in triplicate for each QPCR. The QPCR assay was run three times. EP, electroporation.

To investigate whether electrovaccination affects not only gene expression, but also the persistence of plasmid DNA at the injection site, mice were injected either with 2, 10 or 50 µg pVax-PSA plasmid followed by electroporation or with 50 µg pVax-PSA alone. DNA injection sites were surgically removed 7, 30 or 60 days after DNA administration and the number of persisting plasmid copies in the skin biopsies was determined using qPCR. The group that was not subjected to electroporation had a higher average number of plasmid copies at day 7 compared to mice receiving the same dose followed by electroporation, though the difference was not significant (**Fig. 4**). Plasmid persistence at day 30 or 60 after DNA administration did not differ between mice that received 50 µg DNA by needle injection without electroporation and mice receiving 50 µg DNA together with electroporation (**Fig. 4**). Taken together, the results demonstrate that the number of plasmids persisting in skin decreases over time and that adding electroporation to intradermal DNA administration does not significantly affect the number of persisting plasmid copies in skin.

### Electroporation results in local up-regulation of immune regulatory genes

**Figure 5:**
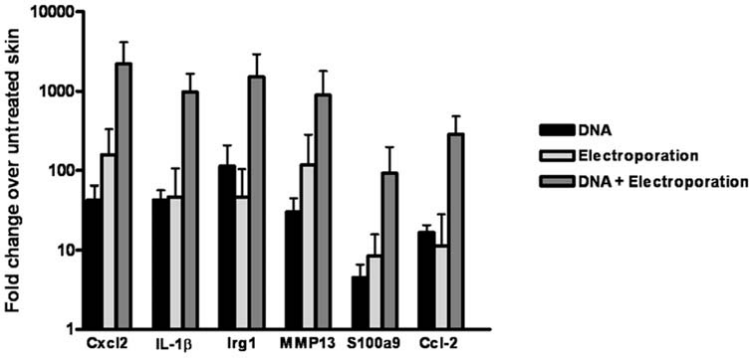
Genes up-regulated at the DNA electrovaccination site. Histogram showing fold increase in gene expression, compared to non-treated control skin, of the indicated genes after the specified treatments. Bars represent mean±SD. The number of independently analyzed samples varied from 4–6; and each sample was run in duplicates or triplicates for each QPCR. The QPCR analysis was run three times.

To investigate the effect of intradermal electroporation on the skin microenvironment, a gene profiling study of the injection site 24 hrs after electrovaccination was performed. No visible differences or signs of inflammation in the skin of the electrovaccinated mice were observed. A gene array analysis demonstrated that the combination of DNA injection and electroporation compared to untreated skin caused an at least 2-fold up- or down-regulation of 436 and 231 genes, respectively (not shown). Based on several pre-processing approaches, a list of ∼500 modulated genes (2 FC and <5% FDR) was analysed using Ingenuity. The highest ranked canonical signaling pathway was the interferon pathway. A gene ontology search of the up-regulated genes showed that among the most up-regulated gene categories are defense responses, immune responses, responses to biotic and external stimulus, inflammatory responses, chemotaxis and MHC class I receptor activity (**supporting information Table S1**). The 20 genes with the highest up-regulation (fold change from 176-25, **Table 1**) included several that encode proteins involved in immune regulation, such as chemokines, markers of cellular (dendritic cells, T cells and keratinocytes) activation, acute phase and proinflammatory molecules. To verify the gene profiling results and to analyze the impact of the two separate components of electrovaccination (DNA injection and electroporation) on the skin microenvironment, skin was examined from 1) untreated mice, 2) DNA injected mice, 3) electroporated mice, and 4) mice that received DNA injection followed by electroporation. A qPCR analysis of the following genes was performed: Chemokine (CXC-motif) ligand 2 (Cxcl2), Interleukin 1 beta (IL-1β), Immunoresponsive gene 1 (Irg1), Matrix metallopeptidase 13 (Mmp13), S100 calcium binding protein A9 (S100a9) and Chemokine (CC-motif) ligand 2 (Ccl2). Expression of all the investigated genes was significantly higher (approximately 10-fold) in skin that had been treated with the combination of DNA and electroporation than in skin that had got either treatment alone (**Fig. 5**).

**Table 1:** Top 20 genes with highest up-regulation in DNA+electroporation treated skin compared to untreated skin.

Gene Symbol	FC	Gene Title
Gsta4	176.4	glutathione S-transferase, alpha 4
Clec4d	175.9	C-type lectin domain family 4, member d
Cxcl2	141.6	chemokine (C-X-C motif) ligand 2
Krt2-6b	99.1	keratin complex 2, basic, gene 6b
Ccl5	97.9	chemokine (C-C motif) ligand 5
Saa3	62.7	serum amyloid A 3
Il1b	47.5	interleukin 1 beta
Cxcl5	41.6	chemokine (C-X-C motif) ligand 5
Il1rl1	40.9	interleukin 1 receptor-like 1
Spp1	39.2	secreted phosphoprotein 1
Irg1	37.5	immunoresponsive gene 1
Chi3l3	33.4	chitinase 3-like 3
Cxcl9	33.3	chemokine (C-X-C motif) ligand 9
Mmp13	31.4	matrix metallopeptidase 13
Slfn3 ///Slfn4	30.3	schlafen 3 /// schlafen 4
Cxcl1	29.5	chemokine (C-X-C motif) ligand 1
Anked1	28.8	ankyrin repeat domain 1 (cardiac muscle)
Rsad2	27.4	radical S-adenosyl methionine domain containing 2
S100a9	25.7	S100 calcium binding protein A9 (calgranulin B)
Cxcl10	25.5	chemokine (C-X-C motif) ligand 10
Ccl2	24.6	chemokine (C-C motif) ligand 2

FC, fold change.

## Discussion

The addition of electroporation to intradermally delivered DNA leads to faster induction of transgene expression, increased levels of produced antigen and up-regulation of genes involved in local inflammatory and immune responses. At the same time, skin electroporation does not increase the number of persisting plasmid copies in skin compared to plasmid injection alone.

Our study shows that, using an *in vivo* imaging system, cells expressing luciferase could be detected as early as 17 min after electrovaccination, while expression of DNA delivered without electroporation was still not detectable 1 hr after vaccination. In similar studies exploring transgene expression in skin after non-electroporative intradermal injection, expression was detectable with an *in vivo* imaging system [26] 10 hrs after DNA administration, and after 6 hrs using the more sensitive *in vitro* detection assay [15]. By collecting and immediately freezing muscle tissue after DNA injection, Doh *et al.* demonstrated that luciferase expression could be detected *in vitro* as early as 30 min post injection (in some mice already after 2 min) [27], showing that even without electroporation, plasmids can be translated within minutes after delivery. However, given a detection limit of around 1000–5000 cells with *in vivo* imaging [28], [29], detection of luminescence within 20–30 min after intradermal electrovaccination in the present study suggests that an exceptionally high number of transfected cells are producing protein shortly after DNA injection. The 2-log increase in gene expression after dermal electroporation in this study is consistent with previous results [15], [16].

Analysis of murine skin sections after DNA electroporation demonstrated that most of the transfected cells were distributed around the panniculus carnosus muscle layer and in the hypodermis. The low number of transfected cells in the epidermis and upper dermis could be a consequence of the difficulty of injecting DNA in these thin skin layers of mice. However, similar studies of intradermal DNA electrovaccination in mice, and larger animal models such as rabbits and pigs, have also shown that the majority of transfected cells are observed within the dermis and subdermal tissues [15], [30], [31], [22]. The transfected cells include adipocytes, fibroblasts, endothelial cells and numerous mononuclear cells with dendritic processes [32]. A different approach, where DNA was applied topically and delivered into murine skin by a tattoo device, demonstrated the presence of transfected cells in both the epidermis and the upper dermis. In contrast to electroporation, tatooing led to a transient β-galactosidase expression, which disappeared over the first 4 days; induction of vaccine specific T cells required three vaccinations within 6 days [33].

In the present study, transgene expression remained high after 60 days and was still detectable after 5 months (data not shown). As the luciferase protein only has a half-life of 3–4 hrs in mammalian cells [34] the detection of luciferase is most probably a result of continuously expressed protein from the plasmid and not of lingering protein expressed months earlier. This long-term expression is not unexpected since others have reported that luciferase is a relatively non-immunogenic protein [25] and its expression can last for 19 months *in vivo* even without electroporation [35]. We therefore mixed luciferase DNA with DNA encoding the more immunogenic prostate specific antigen (PSA) to investigate whether this would affect clearance of DNA transfected cells. We have previously shown that after PSA electrovaccination in mice, PSA-specific CD8^+^ T cells are detectable in peripheral blood at day 11 and peak around day 13 [16]. The gradual clearance of luciferase expression between days 9 and 21 in skin of mice receiving a DNA mixture of luciferase and PSA demonstrates a temporal association between the induction of PSA-specific T cells and the disappearance of cells expressing luciferase. Therefore our data suggest a role for T cells in limiting vaccine antigen expression and indicate that the long-term expression of luciferase is most probably a feature of its low immunogenicity. This hypothesis is further supported by data published by Widera *et al.*, showing unchanged protein expression in immunodeficient mice as opposed to waning expression of a transgene by day 13 in immunocompetent mice [4]. Others confirm these results by demonstrating that loss of vaccine antigen expression in muscle cells became apparent approximately cotemporaneously with the initial detection of antigen-specific T cells to the same immunogen and that persistent vaccine antigen expression was associated with poor vaccine immunogenicity [36]. The elimination of vaccine antigen expressing cells might be especially important for immunotherapy applications, where a long-term, stable expression of the transgene could cause unwanted tolerance. Improvement of the immunogenicity of the vaccine by co-delivery of helper epitopes [2], [37], xenogenic approaches [38], [39], optimized delivery techniques [2], [40] and/or use of adjuvants [40]–[42] is therefore desirable for the generation of robust immunity, while for other applications, such as gene therapy, a long-term, stable expression of the transgene might be beneficial.

The DNA persistence data show that even though luciferase expression is not detectable by *in vivo* imaging 21 days after PSA/luc vaccination, PSA DNA is revealed by PCR at the injection site 60 days after vaccination. This difference might partly be due to the sensitivity of the detection technologies. However, the results might also indicate that cells actively expressing PSA are eliminated while the persisting PSA DNA seen at day 60, could be from cells with extranuclear or intranuclear but transcriptionally inactive plasmid (or parts of plasmids), i.e. from cells containing PSA DNA but that are not expressing the PSA protein. Since the immune response determines the prolonged existence of the antigen expression, it is also possible that multiple vaccinations (boosting inoculations) would have led to a more rapid decline and lower persistence of plasmid copies, especially for the less immunogenic luciferase plasmid. Many pre-clinical studies have shown that electroporation dramatically increases the immune response to DNA vaccines, and several phase I/II trials are currently evaluating electrovaccination in patients [7], [43]. However, since electroporation significantly increases the transgene expression and this probably is due to a higher cellular uptake of DNA molecules, safety-related questions have been raised about the possibility that electroporation could lead to increased DNA persistence and a higher integration frequency [7], [17]. Our results demonstrate a 100-fold increase in antigen expression, but no increase in DNA persistence, after intradermal electroporative DNA delivery. This concurs with recent reports that intramuscular electroporation did not lead to increased plasmid persistence [12], [11]. Studies investigating integration events were not performed after immunization with the PSA plasmid. However, in a parallel study, in which plasmid DNA [44] vectoring viral genes were delivered by electroporation (Cyto Pulse Sciences), we have noted a considerable decrease in plasmid DNA in the skin during 2–3 months following delivery. After one round of Field Inversion Gel Electrophoresis (FIGE), separating the chromosomal DNA from plasmid DNA, a 3 log reduction in plasmid copy number was observed. After the second round of FIGE, the plasmid DNA associated with the genomic DNA was either below the limit of detection (<10 copies/ug genomic DNA) or not quantifiable (<100 copies/ug genomic DNA) as determined by Q-PCR (manuscript in preparation). Furthermore, another study demonstrated that intramuscular electroporation did not result in an increase in plasmid DNA associated with high molecular weight DNA, relative to macaques receiving plasmid DNA without electroporation [11]. However, a study by Wang *et al*. reported on the contrary that intramuscular electroporation did lead to higher levels of plasmid DNA persistence and increased plasmid DNA integration into chromosomal DNA compared to DNA delivery without electroporation [13]. Nevertheless, the frequency of integration was at least 3 orders of magnitude below the spontaneous rate of gene-inactivating mutations [13].

The gene profiling of the treatment site identified several genes encoding proteins involved in immune regulation (such as chemokines, markers of cellular activation, acute phase and proinflammatory molecules) as being highly up-regulated following electrovaccination. The fact that electroporation alone up-regulated several immune/inflammatory related genes supports previous data by others suggesting that electroporation has an adjuvant effect in addition to the increased transfection efficiency [19]. Specifically, the high up-regulation of several chemokines (CXCL2, CCL5, CXCL5, CXCL9, CXCL1 CXCL10) which control the recruitment of leukocytes to the vaccination site is important for the early phases of the immune response [45]. The dramatic up-regulation of glutathione S-transferase (Gsta4), serum amyloid A3 (Saa3), S100a9 and IL-1β further indicates the presence of an inflammatory response [46]. Genes indicating an increased DC presence (Clec4d, IL-1β) [47] and activation (Mmp13) [47] were also up-regulated, which might potentiate the immune response. Even though the gene profiling data support the hypothesis that electroporation has an adjuvant effect, no induction of PSA-specific T cells was detected when electroporation was applied seconds before the DNA injection.

In summary, the addition of electroporation to intradermally delivered DNA leads to faster induction of transgene expression, increased levels of produced antigen and up-regulation of genes involved in local inflammatory and immune responses. Furthermore, application of electroporation after administration of DNA does not increase the number of persisting plasmid copies in skin compared to plasmid injection alone. The information provided in this study is valuable for bringing intradermal electroporation closer to clinical evaluation.

## Materials and Methods

### Animals

Balb/c and C57Bl/6 mice (6–10 wk old) were bred and housed at the animal facility of the Microbiology and Tumor Biology Center at the Karolinska Institutet, Stockholm, Sweden. Mice were anesthetized with 4% isoflurane (Baxter Medical AB, Kista, Sweden) and anaesthesia was maintained at 2–2.5% isoflurane in masks during all intradermal injections, electroporations and live imaging. All experiments using mice were approved by the Swedish National Board for Laboratory Animals.

### Plasmids

The luciferase-encoding plasmid, pVax-luc, and plasmid pVax-PSA encoding prostate specific antigen, have been described previously [16]. The pVax-LacZ plasmid was purchased from Invitrogen (Carlsbad, CA, USA). Plasmids were amplified in *E. Coli* and purified using an Endotoxin Free Plasmid Purification Kit (QIAGEN, Hilden, Germany). The DNA was dissolved in sterile PBS.

### DNA injections and *in vivo* electroporation

Intradermal injections in mice (10–50 µg DNA/20 µl PBS) were made near the base of the tail using a 29 G insulin grade syringe (BD Consumer Healthcare, Franklin Lakes, NJ). Immediately after DNA administration, an electrode was placed over the injection site and voltage was applied (2 pulses, 1125 V/cm, 50 µsec+8 pulses, 275 V/cm, 10 msec). Electrodes had two parallel rows of four 2-mm pins (1.5×4 mm gaps) (Cyto Pulse Sciences, Inc., Glen Burnie, MD). Electroporation was performed using the PA-4000S-Advanced PulseAgile® Rectangular Wave Electroporation System (Cyto Pulse Sciences, Inc.).

### Live imaging of protein expression after DNA vaccination

After DNA injection, mice were injected intraperitoneally with 100 µl/10 g mouse body weight of a 15 mg/ml solution of D-luciferin potassium salt (Xenogen, Alameda, CA) in PBS. Assessment of photonic emissions using the In Vivo Imaging System 100 (Xenogen) was performed 20 min after injection of D-luciferin. Overlay of images and luminescence measurements were made using Living Image software (version 2.50.1; Xenogen). A region of interest (ROI) was manually selected over the signal intensity. The area of the ROI was kept constant for all DNA injection sites and the intensity of luminescence (photons/sec/cm^2^) was recorded within the ROIs. Background luminescence (<200 pixels/sec/cm^2^) was determined by measuring luminescence from mice injected with empty vector DNA (pVAX1) followed by electroporation. For imaging of immediate protein expression, substrate (30 mg/ml D-luciferin) was injected 10 min before DNA injection. Assessment of photonic emissions was started immediately after DNA vaccination.

### β-galactosidase expression

Skin biopsies were removed from female C57Bl/6 mice 24–72 hours after LacZ DNA injection and electroporation. For expression in whole skin, the biopsies were fixed in 4% paraformaldehyde for 3 hours. To detect β-galactosidase activity, the biopsies were incubated in 1 mM X-gal (Invitrogen) at 37°C for 18 hours. For detection of β-glactosidase expression in sections, the biopsies were embedded in OCT; 10 µm sections were fixed in acetone/methanol (50/50) for 10 minutes and subsequently incubated in 1 mM X-gal for 3 hours. Sections were counterstained with eosin and mounted.

### Analysis of IFNγ-producing vaccine-specific CD8^+^ T cells

C57Bl/6 mice were injected intradermally on both flanks with either 10 µg pVax-luc alone or combined with 10 µg pVax-PSA, followed by electroporation. On day 13 after vaccination, 100 µl blood was collected from the tail vein and cells were stained for intracellular IFN-γ production after a 4 hr stimulation with a PSA derived peptide, as previously described [16]. Samples were analyzed using a FACSCalibur flow cytometer (Becton Dickinson) and CELLQuest software (Becton Dickinson).

### DNA persistence in skin – DNA purification and quantitative PCR (qPCR)

Balb/c mice were injected intradermally either with 2, 10 or 50 µg pVax-PSA followed by electroporation or with 50 µg pVax-PSA without electroporation. Mice were sacrificed at day 7, 30 or 60 after injection and the DNA injection sites were surgically removed. The skin biopsies were stored at −80°C until homogenization. Homogenization and total DNA extraction were performed using the DNeasy Blood & Tissue Kit (QIAGEN) according to the manufacturer's instructions. The purified DNA was eluted in 200 µl nuclease-free water (Applied Biosystems, Foster City, CA, USA). The level of plasmid DNA was determined using absolute quantification by qPCR (7500 Real-Time PCR System; Applied Biosystems) according to the manufacturer's instructions with the following PSA-specific primers and probe: 5′-ttgtcttcctcaccctgtcc-3′ (forward primer), 5′-tcacgcttttgttcctgatg-3′ (reverse primer), 5′-FAM-ctcatcctgtctcggattgtg-TAMRA-3′ (probe). The oligonucleotides were purchased from MedProbe (Lund, Sweden) and were used at a final concentration of 300 nM. The qPCR was optimized using standard protocols and the limit of detection of the assay was 10 plasmid copies. The number of plasmid copies per µg genomic DNA was determined using a standard dilution curve of the plasmid in genomic murine DNA. All skin biopsies were processed and analyzed independently.

### RNA isolation and gene expression profiling

C57Bl/6 mice were intradermally injected with 10 µg pVax-PSA followed by electroporation or left untreated. Mice were sacrificed 24 hrs later and the DNA injection sites and control sites were surgically removed. Immediately thereafter, the skin biopsies were snap frozen in liquid nitrogen and stored at −80°C until RNA isolation. The biopsies were homogenized (Polytron, Kinematica) in 1 ml TRIzol (Invitrogen) and total RNA was isolated according to the manufacturer's protocol. All skin biopsies were processed and analyzed independently. The size of the tissues ranged from 45–60 mg. Total RNA was dissolved in 30 µl RNase-free water. Typically, the yield was 40–50 µg of total RNA from 50 mg of tissue. RNA quality was confirmed by analysis in an Agilent 2100 Bioanalyser (Agilent Technologies, Inc, Paolo Alto, CA, USA) and quantified using a Nanodrop. cRNA synthesis and hybridization to U74v2 GeneChip® arrays was done according to the protocol recommended by the manufacturer (Affymetrix Inc., Santa Clara, CA, USA). CEL-files were imported from Affymetrix GeneChip® Operating Software (GCOS), and summary values created using MAS5. Analysis was carried out between the two experimental groups (untreated mice (n = 4), and mice subjected to DNA vaccination followed by electroporation (n = 3)) using significance analysis of microarrays (SAM) [48] which estimates the false discovery rate (FDR). Lastly, a fold change value for treated compared with untreated skin (control) gene expression was applied using the mean of the four replicates for each group. Thus, genes with an FDR of <5% and a mean expression that was at least twofold up- or down-regulated from baseline were considered further. The regulated gene list was consistent with both MAS5 and RMA normalisation (data not shown). For the pathway analysis we utilized a list of ∼500 genes (up- and down-regulated) from the MaS5 and SAM analysis. Pathway analysis was carried out using the web-based bioinformatics tool, Ingenuity pathway analysis (IPA, http://www.ingenuity.com). Affymetrix probe set IDs were uploaded into IPA and queried against all other genes stored in the IPA knowledge database. Probe sets representing genes having direct interactions with genes in the IPA knowledge database are called “focus” genes, which were then used as a starting point for generating functional networks. Each generated network is assigned a score according to the number of differentially regulated focus genes in our dataset. This identified significant canonical pathways, and gene expression changes were annotated using the FC values from the SAM analysis. It should be noted, however, that while the database extends the interpretation beyond mRNA transcript levels (as network genes do not have to be differentially expressed at the mRNA level), the data reflect current knowledge. The raw data files are available at Gene Expression Omnibus (GEO) under data set reference GSE18003.

### Validation of gene array results by qPCR

C57Bl/6 mice were either intradermally injected with 10 µg pVax-PSA followed by electroporation, injected with 10 µg pVax-PSA without electroporation, electroporated only, or left untreated. 24 hrs later the treatment site was excised and total RNA from skin biopsies was extracted as described above and reverse transcribed using TaqMan® Reverse Transcription Reagents (Applied Biosystems). All skin biopsies were processed and analyzed independently. Detection of mRNA was performed using a 7500 Real Time PCR System (Applied Biosystems). Primers were designed using ProbeFinder version 2.35 for the Mouse Universal ProbeLibrary (http://www.universalprobelibrary.com) from Roche Applied Science and were purchased from MedProbe (primer sequences are listed in the **supporting information Table S2**). A pre-optimized primer and probe assay for 18S rRNA (Applied Biosystems) was used as an endogenous control. Primers (300 nM final concentration) were pre-mixed with SYBR®GREEN PCR Master Mix (Applied Biosystems) and applied to 96-well MicroAmp Optical plates (Applied Biosystems). Skin cDNA aliquots were diluted 1∶10 and 3 µl were added in triplicates. Skin cDNA diluted 1∶500 was used for detection of 18S rRNA. Samples were assessed for DNA contamination by including cDNA controls prepared without reverse transcriptase. Standard thermal cycling conditions were applied. For calculation of fold changes of target gene expression between different treatment schedules, the ΔΔCt-method was applied as previously described [49]. The threshold cycle (CT) for 18S was subtracted from the CT for the target gene to adjust for variations in the cDNA synthesis.

### Statistics

Two-way ANOVA analysis was used to investigate differences in luciferase transgene expression between mice receiving electroporation or not. DNA persistence data were analyzed using 2-way ANOVA, where dose/time and electroporation/time were used as variables. Statistical differences in plasmid persistence between groups at day 60 were analyzed using the non-parametric Kruskal-Wallis test. Quantitative PCR data were analyzed using the 2^−ΔΔCT^ method. Normality of q-PCR data was tested using the D'Agostino & Pearson omnibus normality test. As data did not pass the normality test, Kruskal-Wallis tests were used to analyze the impact of treatment on gene expression. P<0.05 was considered statistically significant. All statistical analyses were performed using GraphPad Prism version 4.03 for Windows, GraphPad Software, San Diego California USA, www.graphpad.com.

## Supporting Information

Figure S1 C57Bl/6 mice were injected intradermally on both flanks with 10 µg pVax-PSA. The vaccination site were either not electroporated (+DNA, -EP), electroporated after DNA administration (+DNA, +EP) or electroporated before DNA injection (+EP, +DNA). On day 13 after vaccination, spleens were harvested and cells were stained for intracellular IFN-γ production after a 4 hr stimulation with a PSA derived peptide, as previously described [16].
(0.03 MB TIF)

Table S1 (0.37 MB TIF)

Table S2 (0.07 MB TIF)
