# Supplementary figures and images for: Skin Electroporation: Effects on Transgene Expression, DNA Persistence and Local Tissue Environment
Source: PLoS One. 2009 Sep 30;4(9):e7226. doi: 10.1371/journal.pone.0007226 (PMC2748717; doi:10.1371/journal.pone.0007226)

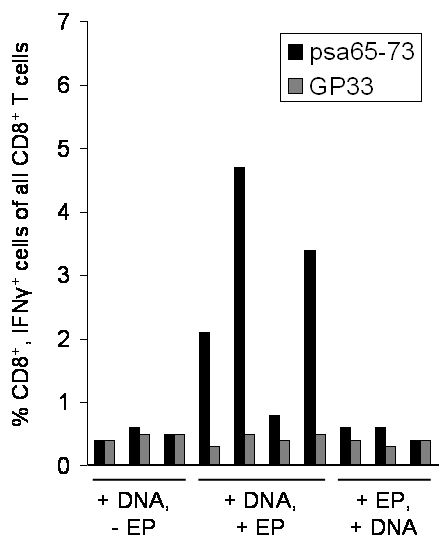

Supplement: Figure S1 — C57Bl/6 mice were injected intradermally on both flanks with 10 µg pVax-PSA. The vaccination site were either not electroporated (+DNA, -EP), electroporated after DNA administration (+DNA, +EP) or electroporated before DNA injection (+EP, +DNA). On day 13 after vaccination, spleens were harvested and cells were stained for intracellular IFN-γ production after a 4 hr stimulation with a PSA derived peptide, as previously described [16]. (0.03 MB TIF) [file pone.0007226.s001.tif]

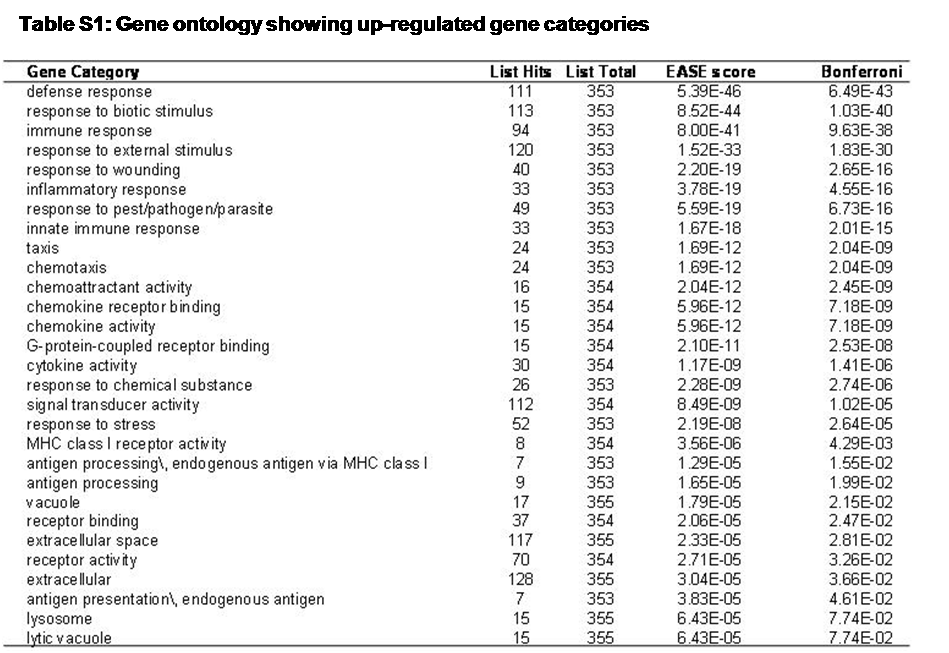

Supplement: Table S1 — (0.37 MB TIF) [file pone.0007226.s002.tif]

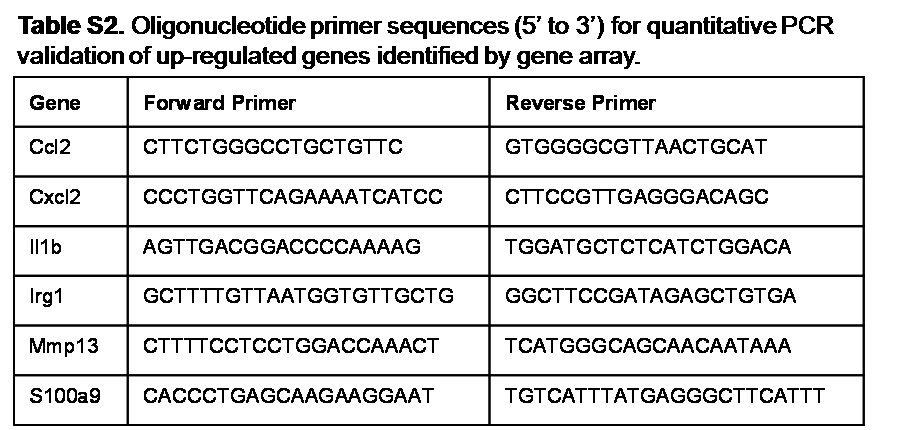

Supplement: Table S2 — (0.07 MB TIF) [file pone.0007226.s003.tif]
